# Necrology

**Published:** 1890-01

**Authors:** 


### NECROLOGY.

THE Committee appointed at the Annual meeting of the U. S. Veterinary Medical Association, held at Brooklyn, N. Y., Sept. 18th, 1889, issued the following prreamble and resolutions upon the death of E. F. Thayer, M.D.V.S., of West Newton, Mass.

WHEREAS, In the death of E. F. Thayer, M.D.V.S., of West Newton, Mass., the veterinary profession of the United States have to mourn the loss of an associate, and one of the founders of their association.

*Resolved*, That by his death we lose the co-operation of one whom we have learned, through long association with, to regard as a large-hearted, liberal minded and conscientious man.

*Resolved*, That, as citizens we feel that the community has lost one whose sterling integrity, faithful discharge of duty and professional ability had rendered his life an ornament to his profession.

*Resolved*, That we tender to his bereaved family our sincere sympathy in their affliction.

*Resolved*, That a copy of these resolutions be forwarded to the family of our deceased associate, and the same be printed in the *American Vetrinary Review* and JOURNAL OF COMPARATIVE MEDICINE AND SURGERY.

By order of the Committee.

JOSIAH H. STICKNEY,

J. F. WINCHESTER,

L. H. HOWARD.

---

WHEREAS, We have learned with regret of the death of Charles C. Moulton, D.V.S., an honored member of the United States Veterinary Association,

*Resolved*, That by his death the veterinary profession lose an associate, valued alike for his strict integrity, faithfulness to the cause of the profession and good fellowship.

*Resolved*, That a copy of these resolutions be spread upon the records of the Asoociation and be pnblished in the *American Veterinary Review* and the JOURNAL OF COMPARATIVE MEDICINE AND SURGERY.

JOSIAH H. STICKNEY,

J. F. MUCHESTER,

L. H. HOWARD.

} *Committee.*

*James Brodie, V.S.*—The following resolutions were acted upon by the Illinois State Veterinary Medical Association, in regular convention assembled, and unanimously adopted :—

SHERMAN HOUSE, CHICAGO, ILL., *Nov. 7, 1889.*

WHEREAS, An all-wise Providence has seen fit to remove from among us our professional brother and former co-laborer, James Brodie, V.S., last of Canon City, Colorado, an enthusiastic and enterprising veterinarian, whose studious and gentlemanly qualities bid fair to place him in the very front rank of our profession ; be it hereby

*Resolved*, That we, the Iliinois State Veterinary Medical Association, deeply lament his loss to the Association and to the profession at large, and that we heartily sympathize with his family in their sad bereavement.

*Resolved*, That these resolutions be spread upon the minutes of this Association; and copies be sent for publication to the *Amurican Veterinary Review* and to the JOURNAL OF COMPARATIVE ANAOMY AND VETERINARY ARCHIVES.

*Resolved*, That a copy of thése resolutions be forwarded to the family of the deceased.

C. E. HOLLINGSWORTH,
*Corrsponding Secretary.*

